# Classical sickle beta-globin haplotypes exhibit a high degree of long-range haplotype similarity in African and Afro-Caribbean populations

**DOI:** 10.1186/1471-2156-8-52

**Published:** 2007-08-10

**Authors:** Neil Hanchard, Abier Elzein, Clare Trafford, Kirk Rockett, Margaret Pinder, Muminatou Jallow, Rosalind Harding, Dominic Kwiatkowski, Colin McKenzie

**Affiliations:** 1 Tropical Metabolism Research Unit, Tropical Medicine Research Institute, University of the West Indies, Kingston, Jamaica; 2 Wellcome Trust Centre for Human Genetics, University of Oxford, Oxford, UK; 3 MRC Laboratories, Fajara, The Gambia; 4 Departments of Zoology and Statistics, University of Oxford, Oxford, UK; 5 Department of Pediatric and Adolescent Medicine, Mayo Clinic, Rochester, USA

## Abstract

**Background:**

The sickle (β^s^) mutation in the *beta-globin *gene (HBB) occurs on five "classical" β^s ^haplotype backgrounds in ethnic groups of African ancestry. Strong selection in favour of the β^s ^allele – a consequence of protection from severe malarial infection afforded by heterozygotes – has been associated with a high degree of extended haplotype similarity. The relationship between classical β^s ^haplotypes and long-range haplotype similarity may have both anthropological and clinical implications, but to date has not been explored. Here we evaluate the haplotype similarity of classical β^s ^haplotypes over 400 kb in population samples from Jamaica, The Gambia, and among the Yoruba of Nigeria (Hapmap YRI).

**Results:**

The most common β^s ^sub-haplotype among Jamaicans and the Yoruba was the Benin haplotype, while in The Gambia the Senegal haplotype was observed most commonly. Both subtypes exhibited a high degree of long-range haplotype similarity extending across approximately 400 kb in all three populations. This long-range similarity was significantly greater than that seen for other haplotypes sampled in these populations (P < 0.001), and was independent of marker choice and marker density. Among the Yoruba, Benin haplotypes were highly conserved, with very strong linkage disequilibrium (LD) extending a megabase across the β^s ^mutation.

**Conclusion:**

Two different classical β^s ^haplotypes, sampled from different populations, exhibit comparable and extensive long-range haplotype similarity and strong LD. This LD extends across the adjacent recombination hotspot, and is discernable at distances in excess of 400 kb. Although the multi-centric geographic distribution of β^s ^haplotypes indicates strong subdivision among early Holocene sub-Saharan populations, we find no evidence that selective pressures imposed by *falciparum *malaria varied in intensity or timing between these subpopulations. Our observations also suggest that *cis*-acting loci, which may influence outcomes in sickle cell disease, could lie considerable distances away from β-globin.

## Background

**Figure 1:**
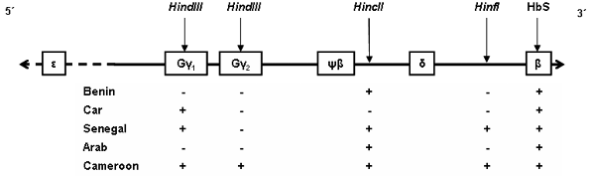
Classical β^S ^haplotypes. The figure illustrates restriction fragment length polymorphisms in a 70 kb region around HBB. Five other *globin *synthesis genes are shown along with the approximate positions of four RFLP sites used to designate the five classical β^S ^haplotypes.

The sickle mutation (β^s^) of the beta-globin locus (HBB), which in the homozygous state gives rise to sickle cell anaemia, is associated with five "classical" haplotypes, each with different geographic distributions across sub-Saharan Africa, Arabia and India [1,2]. These β^s ^haplotypes were first identified by the presence or absence of restriction fragment length polymorphisms (RFLPs) in the 70 kilobases (kb) surrounding HBB [3], and subsequently found to be characterized by strong linkage disequilibrium (LD) across a 'hot spot' of recombination just 5' of HBB [4,5] (Figure 1). The multi-centric geographical distribution of classical β^s ^haplotypes reflects the recency of strong selection pressures imposed by *falciparum *malaria on local human populations [6].

Classical β^s ^haplotypes are named according to their putative geographical origins – Benin, Bantu (Central African), Cameroon, Senegal and Arab. In general, within ethnic groups in which the β^s ^allele has a high frequency, one particular β^s ^haplotype usually predominates; for instance, the Senegal β^s ^haplotype is the most commonly observed β^s ^haplotype in Senegal, although the Benin haplotype is also present [4]. The Benin haplotype is very common (~92%) among the Yoruba in Nigeria [7], and also is common in Jamaica (~71%) [8,9], although Bantu and Senegal types also occur in the Jamaican population [4]. The geographical distribution of classical β^s ^haplotypes has been attributed to independent origins of the β^s ^mutation [1,2] but the role of gene conversion in the transfer of the original mutation(s) between haplotypes has also been discussed [3,4].

Possession of the β^s ^allele in the heterozygous form (HbAS) confers a very strong protection against severe malarial infection compared to mutant (HbSS) and wild-type (HbAA) homozygotes [10-13]. This selective advantage results in a rapid increase in the frequency of the β^S ^allele over several generations in regions of high malarial endemicity. This rise in frequency occurs at a faster rate than meiotic recombination can break down the haplotype background on which the allele first arose. Therefore, like other recently selected alleles such as those found in the *glucose-6-phosphate dehydrogenase *or *lactase *genes [14,15], the β^s ^allele would be expected to maintain its ancestral haplotypic relations over a relatively long genetic distance. We previously used the β^s ^allele as a practical example of recent selection in our description of HAPLOSIMILARITY, a method of evaluating long-range haplotypes [16]. Using 20 high-frequency SNP markers spaced across 414 kb in a sample of Gambian cord bloods we demonstrated a high degree (approximately 60%) of similarity among haplotypes associated with the β^s ^allele [16]. This was considerably higher than haplotype similarity scores for surrounding alleles and also higher than neutral expectations derived from coalescent simulations.

To date, the relationship between classical β^s ^haplotypes and the long-range similarity expected of haplotypes associated with the β^s ^allele has not been defined. A better understanding of classical β^s ^haplotypes is of particular relevance to anthropological and population genetic studies [5,17] and may also be useful for understanding the varying clinical outcomes seen among individuals with sickle cell disease [18,19]. Therefore, we chose to extend the results of our previous study by investigating β^s ^haplotype similarity in two additional populations; specifically asking whether classical β^s ^haplotypes demonstrate strong haplotype similarity over extended physical distances (ie over hundreds of kb). To do so, we analyzed 76 β^s ^chromosomes from Jamaica, identified in a sample of 30 HbSS individuals and a sample of 133 participants from a population survey, as well as 16 β^s ^chromosomes from 60 unrelated Yoruba participants in the International HapMap Project. We then contrasted these results with the analysis of 37 β^s ^chromosomes identified in our previous study of 191 cord blood samples in The Gambia [16].

## Results

### Jamaican β^s ^haplotype similarity

**Figure 2:**
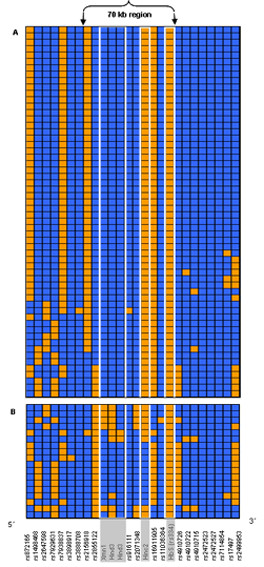
Jamaican β^S ^haplotypes. Haplotypes designated as 'Benin' type are shown in part A; non-Benin type haplotypes are shown in part B. RFLP markers used to distinguish individual β^S ^haplotypes have shaded marker labels and are outlined by the white border. Haplotypes are arrayed along the Y-axis with SNPs on the X-axis. At each SNP position, the major allele of each SNP is represented in blue and the minor allele in orange. For reference, the 70 kb region of the HBB cluster is indicated at the top of the figure.

In the Jamaican population, 26 SNPs met our selection criteria (see Methods) and these were used to construct haplotypes over approximately 200 kb both 5' and 3' of the β^s ^allele (a total of 400 kb – Table 1). Fifty-eight of the 76 β^s ^haplotypes identified (76%) were of the Benin type. Thirty-six (68%) of these Benin haplotypes were identical across 400 kb investigated, and several of the remaining haplotypes differed from the common haplotype at only two or three loci (Figure 2).

**Table 1:** SNPs used for haplotype construction in Jamaicans.

rs number^*a*^	Chr loc^*a*^	Dist from HbS (bp)^b^	Alleles^*c*^	MAF^*d*^
rs872165	5381108	-176300	C/A	0.29
rs1498468	5367607	-162799	T/C	0.33
rs2647598	5332852	-128044	T/C	0.41
rs7929631	5324552	-119744	T/G	0.33
rs7938837	5319683	-114875	C/G	0.28
rs3898917	5284937	-80129	G/T	0.27
rs3888708	5274956	-70148	C/A	0.89
rs2156918	5253468	-48660	C/G	0.23
rs2855122	5233812	-29004	A/G	0.42
Xmn1	5232745	-27937	-/+	0.10
Hind3	5231293	-26485	-/+	0.44
Hind3	5226375	-21567	-/+	0.12
rs916111	5225919	-21111	A/T	0.17
rs2071348	5220722	-15914	A/C	0.10
Hinc2	5217034	-12226	-/+	0.15
rs16911905	5205866	-1058	G/C	0.29
rs11036364	5205580	-772	A/G	0.24
HbS (rs334)	5204808	0	A/T	0.06
rs4910726	5156896	47912	A/C	0.17
rs4910722	5153293	51515	A/G	0.20
rs4910715	5132596	72212	G/T	0.48
rs2472523	5123701	81107	G/A	0.06
rs2472527	5118600	86208	T/C	0.05
rs7114854	5100498	104310	A/C	0.20
rs17497	5014184	190624	G/C	0.44
rs2499953	4967481	237327	A/G	0.26

^*a *^– rs numbers and location on chromosome 6 (Chr loc) are given with reference to HapMap release #20

^*b *^– Negative distances (Dist) from the HbS allele are upstream (5') of the allele, positive distances are downstream (3')

^*c *^– SNP alleles given as major/minor.

^*d *^– Minor allele frequency.

In order to provide a summary statistic for the overall degree of β^s ^Benin haplotype similarity in the population, we used the HS score of the HAPLOSIMILARITY algorithm [16]. The HS score is a measure of the mean similarity of haplotypes calculated by assessing the frequency of distinct haplotypes in smaller, overlapping, sliding windows (see Methods). HS scores range from small values approaching zero (all haplotypes are distinct) to one (all haplotypes are the same). We modified the existing algorithm to determine confidence limits for the estimates of haplotype similarity by bootstrapping the sample of haplotypes 1000 times. The HS score for Jamaican β^S ^Benin haplotypes was 0.689 (95% CI 0.685 – 0.693). By contrast, haplotypes that were identical to the Benin type, as defined by RFLPs, but associated with the major allele (β^A^, N = 93) had an HS score of 0.132 (95% CI 0.131 – 0.133) which was significantly less (P < 0.001) than the score for the β^s ^Benin haplotypes.

We also considered whether the high degree of haplotype similarity among β^s ^Benin haplotypes was simply the consequence of a relatively small number of β^s ^Benin haplotypes having a higher similarity by chance. To do so, we constructed 1000 samples of haplotypes that were not of the β^S ^Benin type (i.e. both β^S ^and β^A ^non-Benin haplotypes); each sample consisted of the same number of haplotypes as the number of β^S ^Benin chromosomes present in the dataset. The mean HS score (HS = 0.133, 95% CI 0.132 – 0.134) of these non-β^S ^Benin haplotypes was significantly lower (P < 0.001) than that obtained for β^S ^Benin haplotypes, suggesting that the high degree of haplotype similarity observed for β^S ^Benin haplotypes was unlikely to be the consequence of sampling error.

### Yoruba β^s ^haplotypes

We wanted to determine whether our observations were unique to the Jamaican population, as well as ascertain the extent to which the strong similarity of β^S ^Benin haplotypes might have been the result of the relatively low marker density employed (one SNP per 16 kb). To do this we utilized SNPs genotyped in Yoruba family trios (the YRI dataset, see Methods) of the International HapMap Project [20].

**Figure 3:**
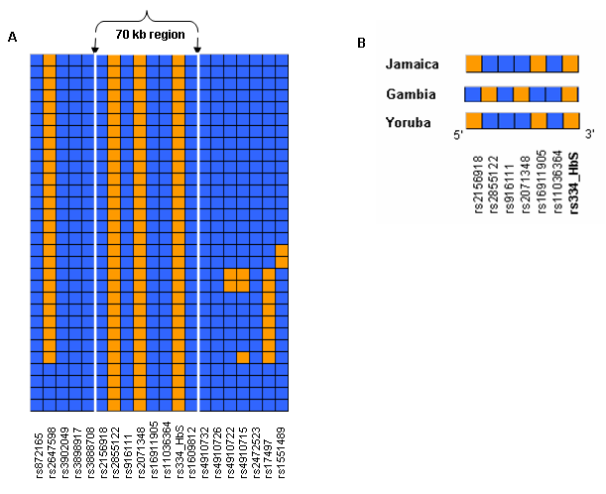
Pattern of HBB LD in Yoruba. The figure shows pairwise D' between the β^S^/β^A ^allele (shaded allele) and 180 high frequency SNPs (minor allele frequency > 0.05). D' > 0.9 is shown in red, D' > 0.7 in green, D' > 0.5 in gray, and values < 0.5 in white. The figure also indicates the 70 kb region around β-globin, and the recombination hotspot (dark gray box).

We first considered parental haplotypes constructed from family trios (see methods) using 181 SNPs spaced across the same 400 kb investigated in the Jamaican sample. Using this increased marker density (≈ 1 SNP every 2 kb), 14 of the 15 β^s ^haplotypes observed were noted to be of the Benin type (see Additional file 1). Again, a high degree of haplotype similarity was noted among the haplotypes (HS = 0.805, 95% CI 0.802 – 0.808), and, as before, this was significantly higher (P < 0.001) than that observed among equivalent samples of non-β^s ^Benin haplotypes (mean HS = 0.221, 95% CI 0.220 – 0.222). Thus, a high degree of β^S ^haplotype similarity appears to be a general feature of β^S ^Benin haplotypes which is independent of both the country of origin and the marker density used to construct the haplotype. This strong haplotype similarity was also reflected in the pattern of LD around the β^S ^allele in this population. As shown in Figure 3, the SNP corresponding to the β^S^/β^A ^allele demonstrated strong LD (mean D' 0.830) with alleles of almost all markers across the 400 kb investigated, and this extended across the 5' recombination hot spot.

Having noted a high degree of haplotype similarity with concomitant strong LD over 400 kb, we then considered the distance over which this might extend. Without *a priori *knowledge of the extent or rate of LD decay along the chromosome, we arbitrarily chose to consider haplotype similarity over a distance of 1 Mb. To do so, an additional 220 SNPs were added from the HapMap YRI dataset, providing a final set of 401 SNPs to cover approximately 500 kb on either side of β^S^. Five of the 14 β^S ^Benin haplotypes were identical across the 1 Mb evaluated, and the haplotypes still exhibited a high degree of similarity (HS = 0.702, 95% CI 0.698 – 0.706).

### Gambian β^s ^haplotypes

**Figure 4:**
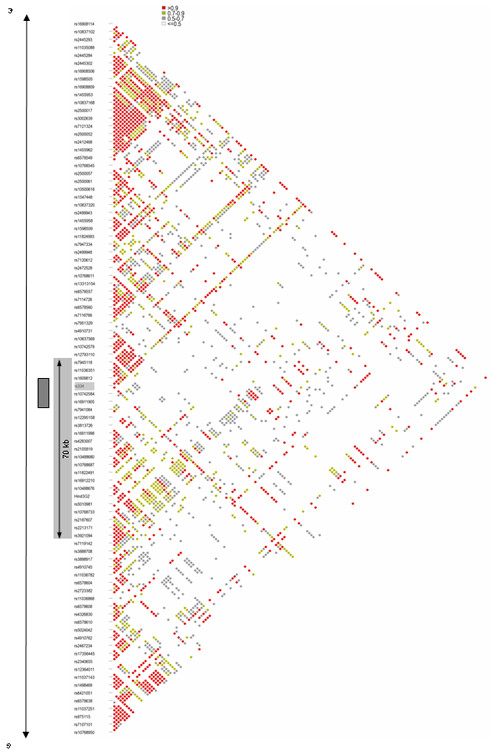
Most common Gambian β^S ^haplotypes. The 'most common' short-range haplotype, including extension of the haplotype to 400 kb is shown in part A. Individual haplotypes are arrayed along the Y-axis with SNPs on the X-axis. At each SNP position, the major allele of each SNP is represented in blue and the minor allele in orange. The 70 kb region defining the 'short-range' β^S ^haplotypes is indicated above the figure and by the white border. A comparison of this 70 kb region in Jamaica, Gambia and Yoruba is shown in part B using markers successfully genotyped in all three populations.

We also re-evaluated the similarity among β^S ^haplotypes analysed in our previous studies in the Gambia [16]. RFLP genotypes were not available in this dataset so we examined haplotypes constructed from the six SNPs genotyped in the 70 kb surrounding the β^S ^allele (see Methods). Using these markers we found that a single haplotype dominated the distribution, comprising 30 of the 37 haplotypes evaluated (81%). Although we were unable to assign this 'most common' haplotype to one of the classical haplotype groups with absolute certainty, it is of a similar frequency to that expected for the Senegal β^s ^haplotype in this population [21]. In addition, when compared to other markers in the same 70 kb region, this 'most common' Gambian haplotype was clearly different from the β^S ^Benin haplotypes observed in the other two populations (Figure 4). These observations, taken together, suggest that this 'most common' haplotype represents the Senegal β^s ^haplotype. Analysis of this 'SNP-defined' Senegal haplotype revealed a high degree of long-range haplotype similarity (HS = 0.827, 95% CI 0.825 – 0.829), wherein 16 (51%) of the haplotypes were identical across the entire 400 kb region (Figure 4).

## Discussion

We have extended our previous observations of long-range β^S ^haplotypes by demonstrating that classical RFLP-defined β^S ^haplotypes are highly conserved over several hundreds of kb. To our knowledge this is the first time that classically described β^S ^haplotypes have been shown to extend over such long genomic distances. We were also able to demonstrate that this conserved β^S ^haplotype similarity was related to a pattern of strong extended LD around the β^S ^allele. Comparable results were found for three different population groups, using differing markers and marker densities. The number of other classical β^S ^haplotypes in the groups sampled was not large enough for us to make definitive statements about all classically-described β^S ^haplotypes; however, since the high degree of haplotype similarity we observed is almost certainly the result of recent selection, and the selective force underlying these observations – severe malaria infection – also applies to other β^S ^haplotypes, it seems likely that a high degree of long-range haplotype similarity will be seen on other β^S ^chromosomes as well. Our findings provide a framework for further investigating the anthropology of the β^S ^allele, including its selection dynamics across the African Diaspora and the origin of classical β^S ^haplotypes, and may have implications for other selected alleles in the genome as well as for the search for genetic modifiers of sickle cell disease.

As an example, Afro-Caribbean and African β^s ^haplotypes have differing demographic and social histories, with concomitant differences in the duration and extent of malarial selection pressure on the allele. The large-scale importation of slaves from Africa to Jamaica some 400 years ago, forcibly moved persons from an area where the selective force in favour of the β^s ^allele was strong – malaria remains a major cause of mortality in equatorial Africa [22], to one where the selective force was substantially less – endemic malaria is not likely to have been a major cause of mortality in Jamaica and was eradicated from the island in 1963 [23,24]. We might then expect differences in malarial selection between African and Afro-Caribbean populations. Similarly, the strong geographic sub-division of β^S ^haplotypes across Africa, which presumably resulted from sub-division of early Holocene sub-Saharan Africans, suggests potential differences in the duration and intensity of malaria among the African population groups sampled as well. These observations suggest that we might have expected significant differences in the degree of haplotype similarity across the three populations.

The HS score in Jamaica was quantitatively less than that in Africa, albeit using differing marker sets and sample sizes. Conversely, a comparison of HS scores generated using only the 16 SNPs that were typed in all three populations, did not demonstrate significant differences between populations (data not shown), despite the expectation of selection differences between the two African groups and even more so between the African groups and the Jamaican sample. This observation is somewhat surprising, although there are likely to be other forces at work which are not accounted for in our appraisal. In the Jamaican population, for instance, there is the potential influence of genetic drift, admixture of sickle haplotypes from across Africa, as well as the non-random survival of individual sickle haplotypes; in our sample the frequency of Benin β^S ^haplotypes among population samples (68%) was similar to that in β^S^β^S ^individuals (78%), albeit with differing sample sizes. Equally, it may be that the differences in the selection pressures themselves are too subtle, too complex, or too recent to affect LD patterns of common SNPs/RFLPs. This would be beneficial for detecting selection from population-based surveys such as the HapMap project, which is already being used as a tool to screen for recently selected alleles in the human genome [25]. A more extensive evaluation of LD/haplotype decay in larger and more diverse datasets with a denser set of markers would help to clarify this.

There remains some uncertainty surrounding the implications of the 5' recombination 'hot spot', both with regard to the origin of the β^S ^allele and to signals of recent selection around HBB. For instance, using sequence data over 5.2 kb, Wood et al [26] found that the recombination hotspot was responsible for attenuation of the haemoglobin C selection signal (HbC); however, strong LD extending over 100 kb and across the β-globin recombination hot spot has also been described in relation to positive malarial selection of the Hemoglobin E (HbE) allele in Southeast Asia [15]. In our dataset, using the Extended Haplotype Homozygosity (EHH) score of the Long-range Haplotype test (LRH) [27], we did not find any substantive differences in haplotype homozygosity between common β^s ^haplotypes 5' of β^S ^and those extending 3' of β^S ^in either Jamaican or Gambian samples (the EHH score is the probability that any two haplotypes extending outwards from a core haplotype or SNP will be the same at a given distance away from the SNP – see Methods). Among the Yoruba, 5' haplotypes appeared to have less similarity and a steeper decline in similarity than 3' haplotypes over 200 kb, but at 1 MB, 5' and 3' haplotypes had comparable degrees of similarity (Additional file 2). The inconsistency of these preliminary results precludes definitive statements about either the impact of the recombination hot spot on the signature of β^S ^selection or the contribution of gene conversion to the origin of classical β^S ^haplotypes; however, a combined approach of short-range sequencing and dense long-range SNP data may help to resolve some of these issues.

Lastly, we offer a note on the potential clinical relevance of our findings. The extent of LD between markers on the haplotypes evaluated may have implications for studies of genetic modifiers of sickle cell disease. To date, such studies have used the strong LD in the surrounding 70 kb to generate hypotheses about local variants that are likely to modulate the HbSS phenotype [18]. Future attempts to identify genetic modifiers of sickle cell disease will have to account for the extended LD observed here, which may require a consideration of cis-acting variants or genes located hundreds to thousands of kilobases away. This approach may provide new candidate loci that either modulate the sickle phenotype or influence traits such as hereditary persistence of foetal haemoglobin, which are known to modify clinical outcomes in the beta-haemoglobinopathies [28].

## Conclusion

We have shown that common β^S ^haplotypes from different populations exhibit a high degree of haplotype similarity, with concomitant strong LD, over hundreds of kilobases despite the adjacent 5' recombination hotspot. To the best of our knowledge, this is the first time that this has been described. These findings suggest little support for differences in selective pressures on β^S ^between major population subdivisions, and may have implications for association studies of genetic modifiers of sickle cell disease in *cis *with the β-globin cluster. Further studies, using both simulated and actual data from multiple populations, are needed to clarify the effects of recombination and population demography on long-range haplotype similarity and LD in the region of this well-established example of a recently selected allele.

## Methods

### Participants

DNA samples from Jamaican adults were obtained by randomly sampling from a population survey that has been described in detail previously [29]. DNA samples from HbSS adults attending the main clinic at the Sickle Cell Unit, University of the West Indies, Jamaica were obtained at random from among samples collected during a previous study of genetic modifiers of HbSS disease [30]. All study samples were anonymised. Use of these samples for the purposes of this study was approved by the University Hospital of the West Indies (UHWI)/University of the West Indies (UWI) Faculty of Medical Sciences Ethics committee. Gambian DNA samples were extracted from a set of cord bloods recruited at the Royal Victoria Hospital, Faraja, The Gambia. Permission for the collection, storage and use of these samples for genetic research was granted by the Joint Gambian Government/MRC Ethics Committee. Cell lines from individuals used in Phase 1 of the HapMap project from the Yoruba in Ibadan, Nigeria (YRI dataset) were obtained from the Coriell Cell Repository at the Coriell Institute for Medical Research [31] as transformed B-lymphocytes from peripheral blood (see Additional file 3 for sample details). Cell lines were cultured and DNA was extracted using CST Genomic DNA Purification Kit [32], and then quantified using NanoDrop technology [33].

### SNP selection and genotyping

In Jamaicans, the ensemble database [34] was used to identify an initial set of 35 SNPs across 414 kb of the β-globin locus on chromosome 11, spanning about 200 kb on either side of the HbS SNP. SNPs were chosen on the basis of validation (preferably in an African-related population), available frequency data, and a desired SNP density of approximately one SNP per 10 kb. Chosen SNPs (including the HbS SNP) were genotyped using MALDI-TOF mass spectrometry (SEQUENOM) on PEP DNA [35] in 137 Jamaican population samples (SNP assay details are available in Additional file 4). SNPs with greater than 10% missing data, genotypes not consistent with Hardy-Weinberg equilibrium (P < 0.01), or minor allele frequencies < 5% were then excluded, resulting in a final set of 22 SNPs. The frequency of the HbS haplotype was 6% in the population sample, which compares favourably with the 5% figure obtained in larger-scale surveys of Jamaicans [36].

Five RFLP sites were additionally genotyped in the same samples using the restriction enzymes Hinf I, Hinc II, Hind III in HbG1, Hind III in HbG2, and Xmn I. Hinf I digests were uninformative as there were multiple Hinf I sites in the amplified PCR fragment; the remaining restriction digests were therefore used to define classically-described β^s ^haplotypes. SNP selection and typing in the 380 Gambian cord blood samples was very similar to the procedure used for the Jamaican samples, and has been described previously [16].

Publicly available SNPs genotyped in the YRI dataset from HapMap release #20 [37] were chosen from a one megabase region spanning first 200 kb and then 500 kb on either side of the β^S ^allele (see Additional file 4). SNPs were chosen on the basis of being polymorphic in the population sampled, having passed HapMap quality control measures, and providing an approximate marker density of one SNP per 2 kb (N = 398). Three RFLP sites – Hind III in HbG1, Hind III in HbG2, and Xmn I (see below)- and the HbS SNP (see above) were independently genotyped in the same samples, for a total of 401 SNPs. Along with SNP rs968857 (which is the same as the Hinc II RFLP), these were used to define classically-described β^s ^haplotypes.

### RFLP genotyping

HBB PCR Primers (see Additional file 5) used to amplify products for RFLP genotyping were designed with careful consideration of the high degree of homology in the region due to gene duplication; this resulted in relatively large amplification fragments. The HBG2 fragment (2734 bp in length) amplified the HBG2 gene and contained restriction sites for both Hind III and Xmn1. The HBG1 fragment (2909 bp in length) amplified the HBG1 gene and contained the restriction site Hind III. The HBB fragment (1200 bp in length) amplified the HBB gene and contained the restriction site Ava II. The recognition site for Hinc II was in an intergenic region with unique flanking sequence, so a small fragment of 118 bp containing it was amplified.

For each PCR reaction 2 μl of genomic DNA at 5 ng/μl was added to 6 μl of PCR mix. PCR mix for 192 reactions was prepared by adding the following: MgCl_2 _(50 mM) – 44 μl; dNTP's (8 mM pool) – 110 μl; ×10 buffer – 110 μl; Biotag 5 U/μl – 5.5 μl; H_2_O – 386.1 μl; 1^st ^PCR primer – 2.2 μl; 2nd PCR primer – 2.2 μl. The PCR mix was the same for all fragments except HBG1 (2909 bp) for which 3.3 μl of each of the forward and reverse primers was used.

PCR protocols for the HBG1 and HBG2 RFLP fragments consisted of an initial five cycle denaturation of 96°C for 1 minute, 94°C for 45 seconds, 62°C for 2.5 minutes, and 72°C for 1 minute; followed by a 29 cycles of 94°C for 45 seconds, 65°C for 2.5 minutes, and 72°C for 1 minute, and a final extension of 72°C for 10 minutes and 15°C for 15 minutes. The PCR protocol for the Hinc2 fragment differed only with regard to the main cycling conditions which required an annealing temperature of 65°C for 45 seconds and an extension temperature of 72°C for 30 seconds. The HBB fragment did not require the initial 5 cycle denaturation; instead 35 cycles consisting of 96°C for 1 minute, 94°C for 45 seconds followed by an annealing temperature of 56°C for 45 seconds, and a 72°C extension for 1 minute was used.

Restriction enzymes and their buffers were ordered from New England BioLabs (Ipswich, MA, USA); digests were carried out according to the manufacturer's recommendations. Digestion products were loaded onto an agarose gel and scored as +/+ if the two alleles were digested, as +/- if one but not the other allele was digested (heterozygote), and as -/- if no digestion occurred in the sample.

### Haplotype construction

In order to improve the integrity of the haplotype inference in the Jamaicans, we omitted any individuals who had more than one site (marker) with missing data, resulting in 133 population samples and 30 HbSS samples. Haplotypes were constructed using the PHASE (version 2.0) software package [38,39]. Among the Yoruba, parental genotypes were first phased using the PHAMILY program [40], which uses parent to offspring transmission to derive phase-known sites from family-trio pedigree data. The resulting haplotypes consisting of phase known and phase unknown sites were then phased using the PHASE algorithm.

### Long-range haplotype similarity

The HS statistic of HAPLOSIMILARITY uses sliding windows to assess the mean similarity of haplotypes (given as the mean of the sum of the squares of the frequencies of distinct haplotypes within a given window) associated with the minor allele of a given SNP. The value of HS ranges from one (all haplotypes associated with the allele are exactly the same) to a minimum given by 1/k_max_, where k_max _is the maximum possible number of distinct haplotypes for a given sliding window size (haplotypes associated with the allele are extremely diverse). We used a sliding window size of ten SNPs (the default option) in our evaluation. HAPLOSIMILARITY (including details on operating characteristics and implementation) is available for public use at the GMAP website [41].

The EHH statistic of the long-range haplotype test (LRH) is very similar to the HS statistic of HAPLOSIMILARITY and is the probability that at a given distance away from a core haplotype or SNP, any two haplotypes extending outward from the core haplotype/SNP will be homozygous at all SNPs. EHH scores range from a minimum of zero to a maximum of one [26].

The Normal approximation for the difference between two proportions [42] was used to test the significance of differences in haplotype similarity between the three populations.

## Authors' contributions

NH was involved in the conception of the study and SNP selection, carried out the statistical analyses and drafted the manuscript. AE and KR were involved in SNP selection and carried out both the RFLP and mass spectrometry genotyping. CT collated and captured the HapMap data. MP and MJ were responsible for recruitment as well as sample and data collection in The Gambia. RH was involved in the conception of the study and helped to draft the manuscript. DK helped in the conception of the study, as well as its design and implementation. CM was involved in the conception of the study, participated in its design and coordination and helped to draft the manuscript. All authors read and approved the final manuscript.

## Supplementary Material

Additional file 1 YRI β^S ^Haplotypes. β^S ^Haplotypes across 400 kb in the HapMap YRI (Yoruba) dataset. HbS is indicated by the black arrow. RFLP markers used to define classical β^S ^haplotypes are indicated in red. At each SNP position, the major allele of each SNP is represented in blue and the minor allele in orange. Click here for file

Additional file 2 β^S ^EHH scores. β^S ^EHH scores of common β^S ^haplotypes extending away from β^S ^in three populations. Negative distances are 5' of the β-globin gene. Positive distances are 3' of the β-globin gene. Click here for file

Additional file 3 YRI sample details. Details of identifiers for Yoruba (YRI) cell lines used to augment HBB genotyping. Click here for file

Additional file 4 YRI markers. Details of Yoruba (YRI) markers used over both 400 kb and 1 Mb, including rs numbers and chromosome location. Click here for file

Additional file 5 Beta-globin PCR primers. 1^st ^and 2^nd ^round PCR Mass Spectrometry primers used for HBB genotyping, including dbSNP and 'rs' reference numbers, as well as links to ensembl contigs. Click here for file

## References

[B1] Nagel RL, Fabry ME, Pagnier J, Zohoun I, Wajcman H, Baudin V, Labie D (1985). Hematologically and genetically distinct forms of sickle cell anemia in Africa. The Senegal type and the Benin type. N Engl J Med.

[B2] Pagnier J, Mears JG, Dunda-Belkhodja O, Schaefer-Rego KE, Beldjord C, Nagel RL, Labie D (1984). Evidence for the multicentric origin of the sickle cell hemoglobin gene in Africa. Proc Natl Acad Sci U S A.

[B3] Orkin SH, Kazazian HH, Antonarakis SE, Goff SC, Boehm CD, Sexton JP, Waber PG, Giardina PJ (1982). Linkage of beta-thalassaemia mutations and beta-globin gene polymorphisms with DNA polymorphisms in human beta-globin gene cluster. Nature.

[B4] Webster MT, Clegg JB, Harding RM (2003). Common 5' beta-globin RFLP haplotypes harbour a surprising level of ancestral sequence mosaicism. Hum Genet.

[B5] Fullerton SM, Harding RM, Boyce AJ, Clegg JB (1994). Molecular and population genetic analysis of allelic sequence diversity at the human beta-globin locus. Proc Natl Acad Sci U S A.

[B6] Rich SM, Licht MC, Hudson RR, Ayala FJ (1998). Malaria's Eve: evidence of a recent population bottleneck throughout the world populations of Plasmodium falciparum. Proc Natl Acad Sci U S A.

[B7] Flint J, Harding RM, Boyce AJ, Clegg JB (1998). The population genetics of the haemoglobinopathies. Baillieres Clin Haematol.

[B8] Wainscoat JS, Bell JI, Thein SL, Higgs DR, Sarjeant GR, Peto TE, Weatherall DJ (1983). Multiple origins of the sickle mutation: evidence from beta S globin gene cluster polymorphisms. Mol Biol Med.

[B9] Antonarakis SE, Boehm CD, Serjeant GR, Theisen CE, Dover GJ, Kazazian HH (1984). Origin of the beta S-globin gene in blacks: the contribution of recurrent mutation or gene conversion or both. Proc Natl Acad Sci U S A.

[B10] Hill AV, Allsopp CE, Kwiatkowski D, Anstey NM, Twumasi P, Rowe PA, Bennett S, Brewster D, McMichael AJ, Greenwood BM (1991). Common west African HLA antigens are associated with protection from severe malaria. Nature.

[B11] Aidoo M, Terlouw DJ, Kolczak MS, McElroy PD, ter Kuile FO, Kariuki S, Nahlen BL, Lal AA, Udhayakumar V (2002). Protective effects of the sickle cell gene against malaria morbidity and mortality. Lancet.

[B12] Jelliffe DB, Humphreys J (1952). The sickle-cell trait in western Nigeria; a survey of 1,881 cases in the Yoruba. Br Med J.

[B13] Allison AC (1954). The distribution of the sickle-cell trait in East Africa and elsewhere, and its apparent relationship to the incidence of subtertian malaria. Trans R Soc Trop Med Hyg.

[B14] Bersaglieri T, Sabeti PC, Patterson N, Vanderploeg T, Schaffner SF, Drake JA, Rhodes M, Reich DE, Hirschhorn JN (2004). Genetic Signatures of Strong Recent Positive Selection at the Lactase Gene. Am J Hum Genet.

[B15] Ohashi J, Naka I, Patarapotikul J, Hananantachai H, Brittenham G, Looareesuwan S, Clark AG, Tokunaga K (2004). Extended Linkage Disequilibrium Surrounding the Hemoglobin E Variant Due to Malarial Selection. Am J Hum Genet.

[B16] Hanchard NA, Rockett KA, Spencer C, Coop G, Pinder M, Jallow M, Kimber M, McVean G, Mott R, Kwiatkowski DP (2006). Screening for recently selected alleles by analysis of human haplotype similarity. Am J Hum Genet.

[B17] Harding RM, Fullerton SM, Griffiths RC, Bond J, Cox MJ, Schneider JA, Moulin DS, Clegg JB (1997). Archaic African and Asian lineages in the genetic ancestry of modern humans. Am J Hum Genet.

[B18] Powars D, Hiti A (1993). Sickle cell anemia. Beta s gene cluster haplotypes as genetic markers for severe disease expression. Am J Dis Child.

[B19] Powars DR, Chan L, Schroeder WA (1990). Beta S-gene-cluster haplotypes in sickle cell anemia: clinical implications. Am J Pediatr Hematol Oncol.

[B20] Altshuler D, Brooks LD, Chakravarti A, Collins FS, Daly MJ, Donnelly P (2005). A haplotype map of the human genome. Nature.

[B21] Currat M, Trabuchet G, Rees D, Perrin P, Harding RM, Clegg JB, Langaney A, Excoffier L (2002). Molecular analysis of the beta-globin gene cluster in the Niokholo Mandenka population reveals a recent origin of the beta(S) Senegal mutation. Am J Hum Genet.

[B22] WHO Africa Malaria Report. http://www.rbm.who.int/amd2003/amr2003/amr_toc.htm.

[B23] Chandler D (1972). Health and slavery: a study of health conditions among  Negro slaves in the Viceroyalty of New Granada and its associated slave trade, 1600-1810.. Doctoral Dissertation, History section, Latin American Library.

[B24] Boyd MF, Aris FW (1929). A malaria survey of the island of Jamaica, BWI. American Journal of Tropical Medicine and Hygiene.

[B25] Voight BF, Kudaravalli S, Wen X, Pritchard JK (2006). A map of recent positive selection in the human genome. PLoS Biol.

[B26] Wood ET, Stover DA, Slatkin M, Nachman MW, Hammer MF (2005). The beta -globin recombinational hotspot reduces the effects of strong selection around HbC, a recently arisen mutation providing resistance to malaria. Am J Hum Genet.

[B27] Sabeti PC, Reich DE, Higgins JM, Levine HZ, Richter DJ, Schaffner SF, Gabriel SB, Platko JV, Patterson NJ, McDonald GJ, Ackerman HC, Campbell SJ, Altshuler D, Cooper R, Kwiatkowski D, Ward R, Lander ES (2002). Detecting recent positive selection in the human genome from haplotype structure. Nature.

[B28] Powars DR, Chan L, Schroeder WA (1989). The influence of fetal hemoglobin on the clinical expression of sickle cell anemia. Ann N Y Acad Sci.

[B29] Cooper R, Rotimi C, Ataman S, McGee D, Osotimehin B, Kadiri S, Muna W, Kingue S, Fraser H, Forrester T, Bennett F, Wilks R (1997). The prevalence of hypertension in seven populations of west African origin. Am J Public Health.

[B30] Haverfield EV, McKenzie CA, Forrester T, Bouzekri N, Harding R, Serjeant G, Walker T, Peto TE, Ward R, Weatherall DJ (2005). UGT1A1 variation and gallstone formation in sickle cell disease. Blood.

[B31] Coriell Institute for Medical Research. http://locus.umdnj.edu/nigms/.

[B32] CST Genomic DNA Purification. http://www.invitrogen.com.

[B33] Nanodrop. http://www.nanodrop.com.

[B34] Ensembl database. http://www.ensembl.org/.

[B35] Griffin TJ, Smith LM (2000). Single-nucleotide polymorphism analysis by MALDI-TOF mass spectrometry. Trends Biotechnol.

[B36] Hanchard NA, Hambleton I, Harding RM, McKenzie CA (2005). The frequency of the sickle allele in Jamaica has not declined over the last 22 years. British Journal of Haematology.

[B37] HapMap project. http://www.hapmap.org/.

[B38] Stephens M, Smith NJ, Donnelly P (2001). A new statistical method for haplotype reconstruction from population data. Am J Hum Genet.

[B39] Stephens M, Donnelly P (2003). A comparison of bayesian methods for haplotype reconstruction from population genotype data. Am J Hum Genet.

[B40] Ackerman H, Usen S, Mott R, Richardson A, Sisay-Joof F, Katundu P, Taylor T, Ward R, Molyneux M, Pinder M, Kwiatkowski DP (2003). Haplotypic analysis of the TNF locus by association efficiency and entropy. Genome Biol.

[B41] GMAP Website. http://www.gmap.net/pub/003.

[B42] Kirkwood BR, Sterne JAC (2003). Essential Medical Statistics.

